# *QuickStats*: Percentage of Injury Deaths* That Occurred in the Decedent’s Home for the Five Most Common Causes^†^ of Injury Death^§^ — United States, 2016

**DOI:** 10.15585/mmwr.mm6726a6

**Published:** 2018-07-06

**Figure:**
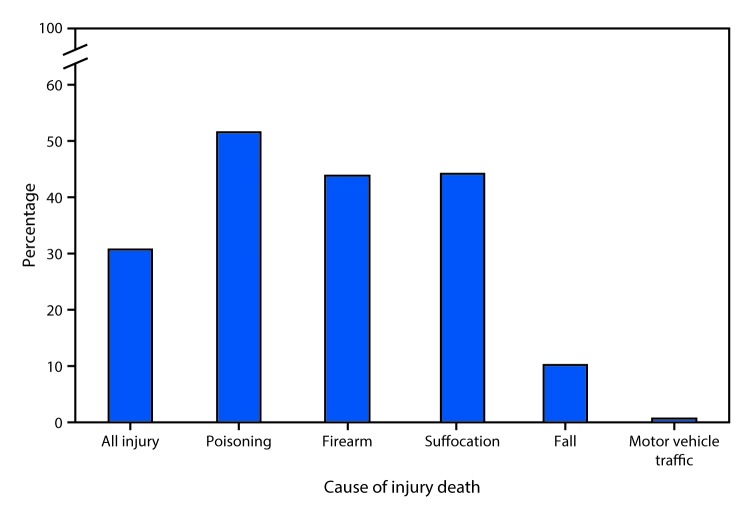
In 2016, 31% of deaths from all causes of injury occurred in the person’s home. The percentage varied by the cause of injury. More than half of the deaths attributable to poisoning (52%) occurred in the home. Approximately 44% of deaths from firearms and suffocation occurred in the home.

**Source:** National Center for Health Statistics, National Vital Statistics System, Mortality File. https://www.cdc.gov/nchs/nvss/deaths.htm.

For more information on this topic, CDC recommends the following link: https://www.cdc.gov/injury.

